# Risk of Depression and Anxiety in Those Who Gave Birth to Children Who Developed Invasive *Group B Streptococcal* Disease: A Population-Based Cohort Study

**DOI:** 10.2147/CLEP.S506809

**Published:** 2025-03-28

**Authors:** Erzsébet Horváth-Puhó, Jaya Chandna, Proma Paul, Claire A Wilson, Henrik T Sørensen, Joy E Lawn

**Affiliations:** 1 Department of Clinical Epidemiology and Center for Population Medicine, Aarhus University and Aarhus University Hospital, Aarhus, Denmark; 2 Maternal, Adolescent, Reproductive & Child Health (MARCH) Centre, London School of Hygiene & Tropical Medicine, London, UK; 3 Department Infectious Disease Epidemiology and International Health, London School of Hygiene & Tropical Medicine, London, UK; 4 Institute of Psychiatry, Psychology and Neuroscience, King’s College London, London, UK; 5 South London and Maudsley NHS Foundation Trust, London, UK

**Keywords:** Streptococcus agalactiae, group B Streptococcus, antidepressant, depression, anxiety, cohort study

## Abstract

**Background:**

Mental disorders such as depression and anxiety are common for women of reproductive age and impact pregnancy and parenting. Invasive *Group B Streptococcus* disease (iGBS) is a leading cause of neonatal morbidity and mortality worldwide. Little is known about the short and long-term risk of common mental disorders in birthing parents whose infants had iGBS in the first 89 days after birth. We aimed to examine the risk of depression and anxiety in birthing parents with iGBS-affected infants in a cohort study with prospectively collected data from Danish registries.

**Materials and Methods:**

Using Danish healthcare registries from 1997 to 2018, we obtained data on iGBS-affected children and their birthing parents. A comparison cohort was randomly sampled (1:50) through risk-set sampling, and matched on persons’ age, year of child´s birth, and parity. The risk of using antidepressant medicines and depression or anxiety diagnosis was analyzed with cumulative incidence function and in Cox proportional hazards regression models.

**Results:**

During the study period, we identified 1,552 women with iGBS-affected child and 76,879 matched comparators. During a median follow-up of 9∙9 years, the cumulative incidence of antidepressants use among birthing parents with iGBS-affected children was 31% (95% confidence interval, CI: 28–34%), as compared with 29% (95% CI: 28–30%) among members of the comparison cohort (hazard ratio 1∙12 [95% CI: 1∙01–1∙25]). A 16% increase in the rate of diagnosed depression or anxiety was observed in the overall follow-up period.

**Conclusion:**

Our findings provide evidence of a slightly increased risk of antidepressant use and diagnosed depression or anxiety in parents who gave birth to children with a history of iGBS compared to a matched cohort of birthing parents whose infants did not develop iGBS. Our findings highlight the importance of addressing the mental health needs of birthing parents affected by their children’ iGBS.

## Introduction

Perinatal mental health (mental health during pregnancy and up to one year postpartum) has major implications for the wellbeing of both the women and her child.^1^ Mental disorders such as depression and anxiety are common for women of reproductive age and impact pregnancy and parenting.^2^ Poor maternal psychological health may be associated with negative attitudes and behaviors toward the child and blunted emotional responses.^3^ Diminished mother-child interaction is strongly associated with poorer neurodevelopmental outcomes, such as behavior and emotional development.^4^

There are well known risk factors for poor maternal mental health, including a previous personal or family history of common mental disorders; experience of violence and abuse; and concurrent adverse life experiences.^5^,^6^ Risk of common mental disorders in the first year after birth may be directly associated with stressful pregnancy events, such as having a preterm birth and/or a baby with severe neonatal illness.^7^,^8^ However, there are a lack of data on long term outcomes for women and later effects on their mental health, particularly for women at high risk who had a neonate with an illness. Recent studies have highlighted the importance of supporting both infants’ physical and developmental needs following neonatal illness, and maternal mental health requirements,^9–12^ especially because the diagnosis and treatment of neonatal illness can be an extremely stressful experience for parents.^8^

Invasive *Group B Streptococcus* disease (iGBS), an infection caused by the bacterium *Streptococcus agalactiae*, is a leading cause of neonatal morbidity and mortality worldwide. Estimates suggest that the burden affects all regions worldwide, with approximately 319,000 infants with iGBS. In high-income countries, mortality rates range from 4% to 10%, and each year, around 40,000 survivors of GBS infection develop moderate or severe neurodevelopmental impairments.^13^ A woman carrying GBS bacteria can vertically transmit GBS during pregnancy or during delivery, which is the primary route of early-onset disease. Infants may also be infected after birth through contact with a GBS carrier. iGBS survivors are at elevated risk of developmental delay, disability, and death.^14–16^ Compared to other neonatal diseases, iGBS may show a stronger association with an increased risk of mental disorders in birthing parents due to its rapid onset, high mortality rate, and its long-term severe complications.

Despite the substantial physical and neurodevelopmental effects in infants with iGBS there is a paucity of research investigating the short- and long-term risk of common mental disorder outcome of birthing parents with iGBS-affected neonates. We have previously analyzed Danish medical and administrative registries to examine long term outcomes for children affected by iGBS, with data linkage for outcomes into the second decade.^14^ The aim of this study was to use national population-based registry data from Denmark^17^,^18^ to investigate the long-term risk of depression and anxiety in those who gave birth to iGBS-affected children compared to those whose child did not develop iGBS.

## Materials and Methods

### Study Design and Participants

This nationwide population-based, retrospective cohort study focused on birthing parents whose children were born in Denmark between January 1, 1997, and December 31, 2018. The study used prospectively collected data from Danish medical and administrative registries. In Denmark, the health care system is tax supported, and provides free universal access to the primary and secondary health care sectors.^18^,^19^ We used the Danish Medical Birth Registry to identify all livebirths in Denmark and their birthing parents. This registry includes every child born in a home or hospital in Denmark, and contains information on both the woman and the newborn.^19^ The Danish Civil Registration System contains individual-level information on all people residing in Denmark, and provides daily updates on vital statistics, including dates of birth, migration and death.^17^ This registry records a unique personal identification number, that was used to link individual-level data across the national registries used in our study.

### Invasive GBS Disease Cohort

The Danish National Patient Registry was used to identify children with iGBS in the first 89 days after birth, including GBS meningitis, sepsis, and pneumonia. The Danish National Patient Registry contains records of all admissions to Danish non-psychiatric hospitals since 1977, and outpatient clinic and emergency room visits since 1995.^20^ iGBS in infants was defined on the basis of discharge diagnoses, according to International Classification of Diseases, 10^th^ Revision (ICD-10) codes. All diagnostic codes used in the study are provided in the supplemental eTable 1. The birthing parents linked to these infants with history of iGBS formed the exposed cohort.

### Matched Comparison Cohort

Each person who gave birth to a child with iGBS was matched with as many as 50 birthing parents according to person’s birth year, year of child´s birth and parity (one, two, or three or more children). The aim of this matching was to address confounding and create a balanced comparison cohort with no history of iGBS. Sampling of comparators was performed randomly with replacement, through risk-set sampling. The index date was defined as the date of iGBS diagnosis in the child. Members of the comparison cohort did not have children with a history of iGBS before their index birth.

### Outcomes

The primary outcome was redemption of prescriptions for antidepressant medicines. The Danish National Prescription Registry, which contains individual-level data on all prescriptions redeemed at outpatient pharmacies since 1995,^21^ was used to obtain information on the redemption of psychoactive medicines. The antidepressant medicines were identified according to the World Health Organization’s Anatomical Therapeutic Chemical (ATC) code system. Prescription codes used in the study are provided in eTable 1.

Secondary outcomes included hospital diagnoses of depression or anxiety, depression alone, anxiety alone, and a composite endpoint of redeemed prescription and discharge depression or anxiety diagnosis. All inpatient and outpatient clinic, primary and secondary discharge psychiatric diagnoses were obtained from the Danish National Patient Registry and Danish Psychiatric Central Research Register.^22^ ICD-10 codes of depression and anxiety are listed in eTable 1.

### Covariates and Other Variables

We obtained data on birthing parents from the Danish Medical Birth Registry, including age at index birth, parity, smoking status during pregnancy, and subsequent deliveries. Body mass index was not available until 2004 and therefore was not included in the analyses. Birthing parent´s health history before the index date was obtained from the Danish National Patient Registry and Danish Psychiatric Central Research Register. Diabetes and alcohol use have a strong confounding relation with both iGBS and poor mental health and were therefore defined as separate covariates. History of common mental disorders, such as depression, anxiety, and antidepressant use, were defined before the index date. Income and highest achieved education, used as proxies of socioeconomic position, were ascertained from Income Statistics Register and Population Education Register provided by Statistics Denmark.^23^ Descriptive data were also utilized on the infant´s sex, prematurity, multiplicity, and neurodevelopmental impairments during follow-up.

### Statistical Analysis

We characterized the exposed iGBS cohort and matched comparison cohort according to person´s characteristics (age at index birth, parity, year of birth, comorbidities, history of common mental disorders, income levels, and smoking habits) and infant characteristics (type of iGBS, sex, prematurity, multiplicity, and neurodevelopmental impairments) by presenting numbers and percentages.

Birthing parents were followed up from the child´s iGBS diagnosis date until the date of an outcome event, death of the birthing person, emigration, or December 31, 2018, whichever occurred first. We calculated the risk of antidepressant use and risk of depression/anxiety separately for birthing parents whose infant had a history of iGBS and the comparison cohort during 0–22 years of follow-up by using cumulative incidence functions that treated death as a competing event. Rates of primary and secondary outcome events per 1,000 person-years (PYs) were computed in the iGBS and comparison cohorts. We used stratified Cox proportional hazards regression models to compare the hazard of common mental disorders among persons with children with history of iGBS and those from the matched comparison cohort. Hazard ratios (HRs) and 95% confidence intervals (CIs) were calculated for both unadjusted and adjusted models. HRs were controlled for matching factors by study design and were adjusted for history of common mental disorders, diabetes, alcohol-related diseases, income, and smoking habits (eFigure 1). The proportional hazards assumption was assessed graphically with log-minus-log plots, and no major violations were detected. Missing data were observed only for confounding variables and were accounted for in the regression analyses as a separate category.

#### Stratified Analyses

We stratified all analyses by the length of follow-up according to the child´s age, as follows: 0–6 years (before school), >6–14 years (preschool and primary education) and >14–22 years (secondary education or higher). In addition, we divided the iGBS cohort into GBS sepsis, GBS meningitis and GBS pneumonia.

We conducted stratified analyses according to birth parent and infant demographic variables: age at giving birth (≤30 years, >30 years), parity (primiparous vs multiparous), birthing parent´s education as an indicator of socioeconomic position, premature birth (<37 weeks, ≥37 weeks of gestational age), the child´s neurodevelopmental impairment during follow-up (yes, no), and presence of a subsequent delivery during follow-up (yes, no). Stratified analyses by ethnicity were not performed due to partial availability of these data.

#### Sensitivity Analyses

We conducted a sensitivity analysis to evaluate the robustness of our main results by excluding persons with a prior redeemed prescription for an antidepressant medicine or a previous depression/anxiety diagnosis.

All analyses were performed in SAS version 9∙4 (SAS Institute, Cary, NC). We followed the reporting guidelines recommended by Strengthening the Reporting of Observational Studies in Epidemiology (STROBE) (eTable 2).

## Results

### Participants and Descriptive Data

Source populations encompassed 1,297,383 live births between January 1, 1997, and December 31, 2017. We identified 1,552 birthing parents whose child developed iGBS, to whom 76,879 comparators were matched. Due to sampling with replacement, 4,912 repeated measurements were included in the comparison cohort.

Characteristics of the birthing parents and infants are presented in Table 1. No differences were observed between cohorts regarding the matching variables (parent´s age, parity, and year of index date), income or median follow-up time. The median age at birth was 30∙4 years (interquartile range [IQR]: 26∙9–33∙6) in both cohorts. Depression and anxiety before the index date were more common in the exposed cohort (5∙7%) than the comparison cohort (4∙8%), as was maternal diagnosis of diabetes (1∙0% vs 0∙5%).

**Table 1:** Characteristics of Birthing Parents Whose Infants Had a History of Invasive GBS Disease and a Matched Comparison Cohort

	Invasive GBS Disease Cohort (N = 1552)		Comparison Cohort (N = 76,879)	
**Birthing parent’s characteristics**				
Birthing parent’s age at index delivery (median, interquartile range)	30∙4 (26∙9–33∙6)		30∙4 (26∙9–33∙6)	
Parity				
1	853	55.0	42,598	55.4
2	401	25.8	20,003	26.0
3+	275	17.7	13,565	17.6
Year of index delivery				
1997–2006	897	57.8	44,315	57.6
2012–2017	655	42.2	32,564	42.4
Comorbidity				
Diabetes mellitus	16	1.0	390	0.5
Alcohol-related diseases	14	0.9	713	0.9
History of common mental disorders				
Depression/anxiety	88	5.7	3,720	4.8
Antidepressant use	155	10.0	7,841	10.2
Income				
Low	403	26.0	19,071	24.8
Medium	771	49.7	38,208	49.7
High	362	23.3	19,261	25.1
Smoking	199	12.8	11,233	14.6
**Infant characteristics**				
Invasive GBS disease				
GBS meningitis	167	10.8	NA	NA
GBS sepsis	1253	80.7	NA	NA
GBS pneumonia	132	8.5	NA	NA
Child´s sex, boys	858	55.3	39,082	50.8
Preterm birth (<37 week)	313	20.2	5,319	6.9
Twin or higher order birth	85	5.5	3,196	4.2
Neurodevelopmental impairment during follow-up	233	15.0	5,612	7.3

**Notes**: Numbers are presented as n (%) unless otherwise indicated. Missing data was observed for parity (1∙5% in iGBS cohort vs 0∙9% in comparison cohort), maternal income (1∙0% in iGBS cohort vs 0∙4% in comparison cohort), maternal smoking (14∙1% in iGBS cohort vs 12∙7% in comparison cohort), and for child´s sex (0∙01% in comparison cohort).

Most infants with iGBS (80∙7%) were classified as having GBS sepsis, whereas 167 (10∙8%) infants were diagnosed with GBS meningitis, and 132 (8∙5%) had GBS pneumonia. Infants in the iGBS cohort were more likely to be male (55∙3% vs 50∙8%) and to be born prematurely (20∙2% vs 6∙9%) than infants in the comparison cohort. Infants in the exposed cohort had more neurodevelopmental impairments during follow-up than those in the comparison cohort (15∙0% vs 7∙3%).

### Primary and Secondary Outcomes

During a median follow-up of 9∙9 years (IQR: 5∙0–16∙7 years), 368 (23∙7%) birthing parents with a child with a history of iGBS used antidepressants [rate per 1,000 PY 22∙7 (95% CI: 20∙4–25∙0)], as compared with 16,956 (22∙1%) persons in the comparison cohort [rate per 1,000 PY: 20∙6 (95% CI: 20∙3–20∙9)]. A total of 163 (10∙5%) birthing parents with a child with iGBS [rate per 1,000 PY: 8∙67 (95% CI: 7∙34–10∙0)] and 6,912 (9∙0%) members of the comparison cohort [rate per 1,000 PY: 7∙37 (95% CI: 7∙20–7∙54)] were diagnosed with depression or anxiety during follow-up (Table 2).

**Table 2:** Rates and Hazard Ratios During 0–22 years of Follow-up, Comparing the Risk of Common Mental Disorders in Those Who Gave Birth to an Infant With a History of Invasive GBS Disease vs a Matched Comparison Cohort of Those With an Infant Without History of Invasive GBS Disease

	Number of Outcome Events		Rate per 1,000 PY (95% CI)		Hazard Ratio (95% CI)	
	Invasive GBS Disease Cohort	Comparison Cohort	Invasive GBS Disease Cohort	Comparison Cohort	Unadjusted	Adjusted
**Primary outcome**						
Antidepressant use	368	16,956	22∙67 (20∙35–24∙98)	20∙60 (20∙29–20∙91)	1∙12 (1∙01–1∙24)	1∙12 (1∙01–1∙25)
**Secondary outcomes**						
Depression or anxiety diagnosis or use of antidepressants	401	18,572	25∙00 (22∙56–27∙45)	22∙89 (22∙56–23∙22)	1∙11 (1∙00–1∙22)	1∙12 (1∙01–1∙23)
Depression or anxiety	163	6,912	8∙67 (7∙34–10∙00)	7∙37 (7∙20–7∙54)	1∙18 (1∙01–1∙38)	1∙16 (0∙99–1∙36)
Depression	71	3,357	3∙65 (2∙80–4∙50)	3∙48 (3∙36–3∙60)	1∙04 (0∙83–1∙32)	1∙01 (0∙79–1∙28)
Anxiety	123	5,164	6∙42 (5∙29–7∙56)	5∙42 (5∙27–5∙57)	1∙19 (0∙99–1∙42)	1∙18 (0∙98–1∙41)

**Figure 1:**
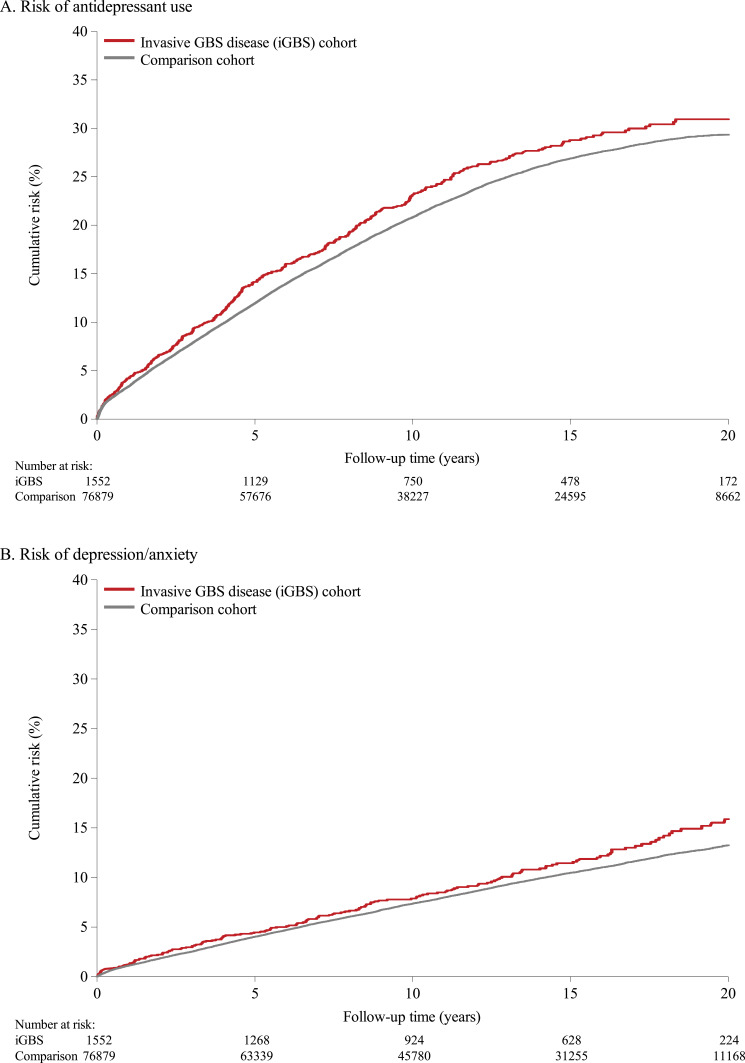
Risks of antidepressant use (**A**) and depression/anxiety (**B**) in those who gave birth to children who developed invasive GBS disease and members of the matched comparison cohort.

The cumulative risk curves of antidepressant use and the risk of depression or anxiety during 0–22 years of follow-up are presented in Figure 1. The adjusted HR of antidepressant use was 1∙12 (95% CI: 1∙01–1∙25), and a 16% increase in the risk of diagnosed depression/anxiety was observed in the overall follow-up period (adjusted HR 1∙16 [95% CI 0∙99–1∙36]; Table 2).

### Stratified Analyses

The analyses stratified by follow-up time are presented in Table 3. The adjusted HR for antidepressant use was 1∙18 (95% CI: 1∙03–1∙34) in the first 6 years of follow-up and declined thereafter. The adjusted HR was 1∙02 (95% CI: 0∙85–1∙22) in the >6–14-year period and 1∙03 (95% CI: 0∙86–1∙24) in the >14–22-year period. Although we observed a small increase in the risk of diagnosed depression or anxiety in the 0–6 year and >6–14 year follow-up periods (adjusted HR: 1∙08 [95% CI: 0∙85–1∙36] and 1∙11 [95% CI: 0∙86–1∙44], respectively), the risk substantially increased during >14–22 years of follow-up, and the adjusted HR was 1∙63 (95% CI: 1∙13–2∙33).

**Table 3:** Rates and Hazard Ratios Comparing the Risk of Common Mental Disorders in Those Who Gave Birth to an Infant With a History of Invasive GBS Disease Vs a Matched Comparison Cohort of Those With an Infant Without History of Invasive GBS Disease, by Follow-up Time

	Number of Outcome Events		Rate per 1,000 PY (95% CI)		Hazard Ratio (95% CI)	
	Invasive GBS Disease Cohort	Comparison Cohort	Invasive GBS Disease Cohort	Comparison Cohort	Unadjusted	Adjusted
Antidepressant use						
0–6 years	231	9,983	29∙57 (25∙75–33∙38)	25∙43 (24∙93–25∙93)	1∙16 (1∙02–1∙32)	1∙18 (1∙03–1∙34)
>6–14 years	118	6,042	19∙24 (15∙77–22∙71)	19∙36 (18∙87–19∙85)	1∙04 (0∙86–1∙24)	1∙02 (0∙85–1∙22)
>14–22 years	19	931	8∙28 (4∙56–12∙00)	8∙08 (7∙57–8∙59)	1∙03 (0∙86–1∙24)	1∙03 (0∙86–1∙24)
Depression or anxiety diagnosis or use of antidepressants						
0–6 years	246	11,031	31∙77 (27∙80–35∙73)	28∙36 (27∙83–28∙89)	1∙12 (0∙98–1∙27)	1∙14 (1∙00–1∙30)
>6–14 years	130	6,403	21∙49 (17∙80–25∙19)	20∙87 (20∙36–21∙38)	1∙07 (0∙90–1∙28)	1∙07 (0∙90–1∙27)
>14–22 years	25	1,138	11∙14 (6∙77–15∙51)	9∙84 (9∙26–10∙41)	1∙18 (0∙79–1∙77)	1∙16 (0∙78–1∙73)
Depression or anxiety						
0–6 years	73	3,360	8∙76 (6∙75–10∙77)	8∙09 (7∙82–8∙36)	1∙08 (0∙86–1∙37)	1∙08 (0∙85–1∙36)
>6–14 years	59	2,604	7∙93 (5∙91–9∙95)	7∙03 (6∙76–7∙30)	1∙13 (0∙87–1∙46)	1∙11 (0∙86–1∙44)
>14–22 years	31	948	10∙23 (6∙63–13∙84)	6∙24 (5∙84–6∙64)	1∙66 (1∙16–2∙37)	1∙63 (1∙13–2∙33)

Having offspring with GBS meningitis was associated with an elevated risk of antidepressant use among birthing parents during the overall follow-up period (adjusted HR 1∙25 [95% CI: 0∙90–1∙73]). This risk was lower for those whose infants had GBS sepsis (adjusted HR 1∙10 [95% CI: 0∙98–1∙24]) and GBS pneumonia (adjusted HR 1∙17 [95% CI: 0∙83–1∙64]). GBS sepsis and pneumonia were associated with an elevated risk of diagnosed depression or anxiety (adjusted HRs 1∙17 [95% CI: 0∙98–1∙39] and 1∙46 [95% CI: 0∙90–2∙38], respectively; eTable 3).

We observed a similar pattern of findings among birthing parents with preterm and term births, in the two age groups, and those with and without subsequent deliveries during follow-up. The increase in the risk of antidepressant use and depression/anxiety was higher in multiparous persons (adjusted HR 1∙17 [95% CI: 1∙01–1∙37] and 1∙36 [95% CI: 1∙09–1∙69], respectively). Across parental education subgroups, we observed the strongest associations between low education and antidepressant use (adjusted HR: 1∙29 [95% CI: 1∙11–1∙49]) or diagnosed depression/anxiety (adjusted HR: 1∙14 [95% CI: 0∙91–1∙44]) (eTable 4).

### Sensitivity Analyses

Sensitivity analyses excluding birthing parents with a prior redeemed prescription for an antidepressant medicine, or a history of depression/anxiety did not indicate substantial effects on the risk estimates for the primary or the secondary outcomes (eTable 5).

## Discussion

Our study indicated that having a child with a history of iGBS was associated with a slightly higher risk of antidepressant use and diagnosed depression or anxiety among birthing parents than observed in the matched comparison cohort. Previous research has focused primarily on the physical and neurodevelopmental effects in affected infants, while neglecting maternal mental health needs. This study addressed this research gap by providing insights into the long-term psychological consequences experienced by these birthing parents. We found that one in four birthing parents whose child had a history of iGBS had been diagnosed with depression/anxiety or had used antidepressants compared to one in five birthing parents whose child did not develop iGBS.

Our results are consistent with those from a meta-analysis reporting a 75% greater risk of depression and 40% greater risk of an anxiety disorder in parents (mostly women) of chronically ill children than healthy children.^24^ Another study from the United Kingdom highlighted an association between children´s life-limiting conditions and increased referral to mental health services in women.^25^ However, the associations were modest, and varied depending on the length of follow-up and the type of GBS disease. Two Danish studies demonstrated that giving birth to a child with a major birth defect increased mortality and risk of new-onset maternal psychiatric disorders when compared to matched comparators without a child with iGBS.^12^,^26^

We observed an increase in the risk of antidepressant use from early on, in the first 6 years after an iGBS episode, which then seemed to decline. This finding might have been because psychological distress, due to hospitalization and uncertainty of the disease outcome, was initially experienced but subsequently improved after recovery.

When stratified by follow-up time, a surprising finding was that the risk of diagnosed depression or anxiety was highest in the later follow-up period, during >14–22 years of follow-up. A Dutch study has reported that 17% of women with a child with meningococcal septic shock reported emotional problems in a follow-up study 2 years after pediatric ICU discharge, but no differences were found in HR-QoL scores with respect to those in the normative Dutch population, thus suggesting that child health has no negative effects on parents and families. However, this study had a relatively short follow-up time and did not control for previous health history.^27^ Thus, the long-term psychological effects of having an infant with iGBS may manifest more prominently in later years. Among women with a child with severe meningococcal disease being treated in the pediatric ICU, improvements in mental health were observed at 36 months^28^ or more after infection. In another study, improvements in health-related quality of life have been observed after median 10 years after discharge.^29^ An alternative explanation for the observed association could be the limited data available during this later follow-up period or a potential artifact of the modeling process.

Our study also explored associations between different types of GBS disease and common mental disorders in birthing parents. Those persons whose infants had GBS meningitis were found to be at a higher risk of antidepressant use and diagnosed depression or anxiety than birthing parents with an infant with GBS sepsis or pneumonia. In a study of women with an infant surviving invasive meningococcal disease, 40% have been found to have mental distress scores, and the risk of maternal distress has been found to be significantly greater in women with a child treated in pediatric ICUs.^30^ These findings highlight the potential differential effects after meningitis and are likely to be mediated by the higher risk of impairment^16^,^31^ after GBS meningitis.

Strengths of this study include its large sample size, virtually complete long-term follow-up, and the use of population-based registries, which provided comprehensive and valid data with matching which helped address potential confounding factors. The stratified analyses allowed for further exploration of the associations according to follow-up time and demographic variables.

However, there are also limitations to consider. The study relied on registry data, which might have inherent limitations, such as potential misclassification or incomplete information. Identification of iGBS was based on ICD codes rather than a microbiologically confirmed diagnosis, and therefore some children who did not have culture confirmed iGBS might have been included in the iGBS cohort, or children with iGBS who were not assigned the correct ICD code might have been missed. Dispensed medication data in the Danish Prescription Registry are highly valid, however lack information on adherence and over-the-counter medications.^21^ The positive predictive values for depression range between 65% for mild depression and 83% for severe depression in the Danish registry of discharge diagnoses.^32–34^ Detailed information on individual-level psychosocial factors, such as social support or coping strategies, were not available, but could have influenced mental health outcomes. We did not observe a difference in the rates of antidepressant use and depression/anxiety diagnosis for birthing parents in the iGBS cohort over the entire follow-up period, regardless of the child’s neurodevelopmental impairment diagnosis. Given that the neurodevelopmental impairment diagnosis occurred during follow-up, we were unable to determine whether antidepressant prescription or the depression/anxiety diagnosis occurred first; therefore, we were unable to explore the potential mediating effect of the child’s neurodevelopmental impairment on common mental disorders in birthing parents. Stratified analyses by race/ethnicity were not performed in this study due to partial availability of these data. This study focused on birthing parents in Denmark; consequently, the generalizability of our findings to other populations or healthcare settings may be limited.

## Conclusions

In conclusion, this population-based cohort study provides evidence of a slightly increased risk of antidepressant use and diagnosed depression or anxiety among parents who gave birth to children with a history of iGBS compared to a matched cohort of birthing parents whose infants did not develop iGBS. The associations were modest and varied depending on the length of follow-up and the type of GBS disease. Our findings underscore the importance of addressing the mental health needs of birthing parents whose infants have invasive GBS disease, particularly over the long term. Further research is needed to explore the wider experiences of this population, underlying mechanisms to design and implement interventions to support the long-term psychological well-being of these birthing parents and to assess the risk of common mental disorders in non-birthing parents.

## Abbreviations

iGBS, Invasive *Group B Streptococcus* disease; ICD-10, International Classification of Diseases, 10^th^ Revision; ATC, Anatomical Therapeutic Chemical; PYs, Person-years; HRs, Hazard Ratios; CIs, Confidence Intervals; STROBE, Strengthening the Reporting of Observational Studies in Epidemiology; IQR, Interquartile Range.
